# Areca nut use among adolescents: How do we prevent and control this problem?

**DOI:** 10.7189/jogh.13.03022

**Published:** 2023-05-12

**Authors:** Nilesh Chatterjee, Himanshu A Gupte

**Affiliations:** Narotam Sekhsaria Foundation, Mumbai, Maharashtra, India

**Figure:**
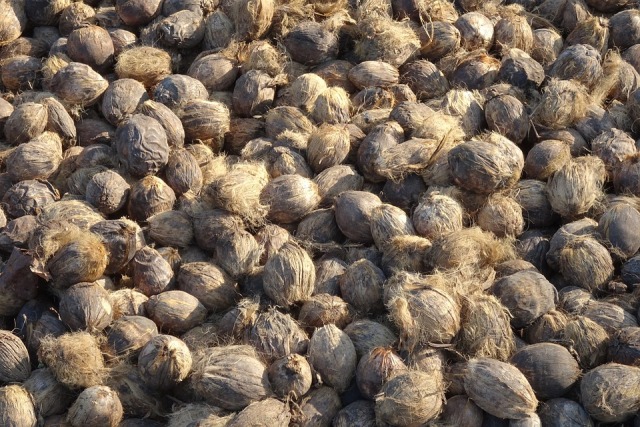
**Photo:** Areca nut. Source: Pixabay, available: https://pixabay.com/photos/areca-nut-betel-nut-fruit-harvested-4033593/, free to use under the Content License.

Areca nut consumption, like tobacco and alcohol use, is a major risk factor for oral cancer. Areca nut is the primary ingredient in betel quid, prepared by adding slaked lime, betel leaf and sometimes tobacco. It is also used in “mawa” with tobacco and slaked lime, in commercial products such as gutkha and pan masala, and marketed in cheap colourful sachets with added flavours and sweeteners [1-4]. Areca nut, used with or without tobacco, has been evaluated by the International Agency for Research on Cancer as a group 1 carcinogen with large-magnitude risks reported in studies comparing betel quid chewers and never users [1]. A recent (2022) meta-analysis, based on 45 case-control studies and eight cohort studies, assessed the association of areca nut with oral cancer and found a pooled adjusted relative risk of 7.9 (95% CI = 7.1-8.7). The risk of oral cancer increased in a dose-response manner with the amount consumed and the number of years of chewing. Approximately half of oral cancers reported in India and Taiwan are attributed to betel quid chewing with a population attributable fraction of 53.7% for Taiwan and 49.5% for India, a disease burden that is preventable [4]. Oral leukoplakia and oral submucous fibrosis are oral potentially malignant disorders caused by areca nut chewing that can progress to oral cancer with continued use [2-4]. Areca nut has also been reported to cause or exacerbate myocardial infarction, hepatotoxicity, obesity, type II diabetes, and asthma [2].

Areca nut is the fourth most commonly abused addictive substance after nicotine, alcohol and coffee [2-5]. It contains chemicals such as arecoline, guvacine, arecaidine and guvacoline, which are responsible for both carcinogenicity and addictiveness. Arecoline is a weak activator of the same brain receptors that cause nicotine addiction; it primes users for the addicting effects of nicotine and the addition of tobacco to existing areca nut use. Physical dependence, which leads to withdrawal symptoms, comes from the nicotine-like activity of arecoline; several other brain receptors, including those targeted by narcotic drugs, are also affected [2-4].

Areca nut is consumed in various forms - raw, processed by boiling, roasting, fermenting, and with or without tobacco. It is used mostly in countries in the Asia-Pacific region such as India, Pakistan, Nepal, Bangladesh, Myanmar, Sri Lanka, Taiwan, Cambodia, Malaysia, Papua New Guinea, Pacific island nations and worldwide among emigrants of these countries [2-5]. Areca nut chewing is considered a culturally acceptable behaviour across social groups in these countries, integrated into daily life and ceremonial situations, and freely shared with family members. While the number of countries affected are few, the absolute numbers of users are reported to be more than 600 million. In India alone, 224 million individuals consume areca nut; and 18% of 15-18-year olds and 21% of 19-23-year olds reportedly use areca nut [6].

Areca nut represents the perfect storm of “substance abuse today and cancer tomorrow”, especially in demographically younger and populous countries in the global south. There is unrestricted use by children and adolescents in South Asian countries and the Pacific islands [3,6]. It is also considered a gateway drug for later use of tobacco and other addictive substances [7]. Adolescence is a time of transition in the life-course. Adolescents become curious and experiment, and some of that risk-taking carries over into initiation of substance use and addiction.

Areca nut is addictive, easily available and culturally acceptable in many of these countries. Studies have found that while adolescents are wary of tobacco and alcohol use owing to higher harm perceptions, areca nut consumption is perceived as low-risk and a harmless past-time [8]. Adolescents perceived tobacco as risky because of greater exposure to messaging on harms through mass-media, interpersonal networks including parents, and classroom health-education sessions. Unlike tobacco, there is inadequate knowledge about the harmful consequences of areca. Despite being a cancer-causing substance, adolescents perceived areca nut as harmless because commercially-marketed products were sweetened and had a fresh after-taste compared to bitter-tasting smokeless tobacco products. Adolescents argued, how can something so sweet and flavourful be harmful [8]? Furthermore, sports, which boosts adolescents’ health and self-esteem, and often the policy-makers’ favoured deterrent to substance use among youth, was found to facilitate areca nut use. Adolescents, who used areca, said that it gave them a physical boost and energy while playing physical sports, while running to fetch a ball, batting, or bowling as they played cricket [8]. Research has also found that areca nut use, as compared to tobacco, was more prevalent among adolescent girls owing to greater cultural acceptability and seeing other women in the family use it [8,9].

Understanding areca nut use among adolescents and the underlying psychosocial context requires an examination of who initiates use and why? What factors influence initiation of areca nut use: academic non-performance, peer groups, lack of friends, lack of hobbies, parental influence, cultural acceptance, easy availability, or other factors? A deeper behavioural and cultural understanding of areca use among adolescents is required. Evidence-based practices for prevention and cessation are scarce [5]. Where adolescent psychosocial cessation programmes for areca nut exist, especially in the Indian context, they are combined with tobacco cessation due to resource and time constraints. This is defensible on the grounds that arecoline neuro-pathway is similar to that of nicotine. However, our field experiences provide slightly paradoxical findings. Existing users of tobacco often step down to areca as a substitute to aid their tobacco-cessation efforts. Clubbing areca with tobacco in psychosocial cessation interventions could be helpful to this type of user. However, those using areca-only are often different, likely to be female or younger and often regard areca as harmless and tobacco as harmful. The consequences of mixing this type of areca-user with the tobacco-user in one cessation group need more in-depth study. Areca use has very different cultural, social and psychological antecedents as compared to tobacco. The lack of recognition of its harms to health and absence of deterrents in the form of high pricing, restrictions, prevention messaging has probably enabled areca use to become an epidemic in large parts of the global south [2,5]. Creating a healthier future in affected countries includes the elimination of areca nut use among adolescents. This needs more research to understand adolescent risk-taking behaviours and addictions within their cultural context, and exploring meaningful, healthy alternatives in the educational, sports, leisure or life-skills spheres.

What is the future of action against areca nut? Local-level actions led by physicians, dentists, community-based organizations, or concerned citizens could spur local movements against areca nut. However, this might remain limited to certain geographies and be difficult to scale up. Areca nut is not just historically and culturally accepted, but a major revenue-generating crop, which makes policy makers wary of red-flagging it as a public health problem. Galvanising wide public and national-level government action against areca nut will need a multi-level, multi-channel campaign including mass media and community mobilisation to raise awareness, set agendas, and challenge social norms; and advocacy campaigns to influence politicians, policy and decision-makers, and health professionals. It is generally difficult to stimulate bold national-level health policy-change, especially in these affected countries in the global south. The health investments are generally low and decision-makers cite more pressing priorities such as poverty, hunger-reduction, malnutrition, infectious diseases, and child mortality. There is little time and energy left for adolescent health concerns, especially addictions or long-term non-communicable diseases such as cancer.

Furthermore, no internationally accepted policy or framework exists for the control of areca nut despite the risks it poses [3,5]. The World Health Organization’s (WHO) Framework Convention on Tobacco Control provided evidence-based policies for reducing tobacco use and stimulated policy-makers to implement national-level policies, government regulations and taxation, which helped reduce tobacco use. Regional efforts could facilitate a similar framework for areca nut control. Civil society organisations and academic institutions from affected countries and the national or regional offices of international agencies such as WHO and United Nations Children’s Fund (UNICEF) could come together to create the much-needed platform and framework for areca control. This collective voice could help promote research, action and cooperation at multi-lateral, bilateral, or national levels. This platform could help in setting up collaborative, multidisciplinary, multi-country research projects. It could help advocate and influence governmental investment in mass media or social media campaigns, crucial to bring areca nut harms to public attention. Virtual knowledge management centres can curate empirical and tacit evidence that already exists for areca nut prevention. Periodic meetings and seminars engaging multi-sectoral stakeholders will facilitate advocacy efforts. These meetings could help bring academics, researchers, and activists from the global north to provide relevant expertise. Regional and national tobacco programmes are already in place in these countries. Activating such tobacco-control platforms and enrolling tobacco activists and researchers to the cause of areca nut prevention and control could help greatly [10]. Areca nut use stands at the intersection of biology, history, culture, society, economy and various interpersonal and intrapersonal factors. Controlling this neglected, “wicked” problem of areca nut calls for a multi-disciplinary, multi-sectoral, multi-country health response in which professionals, policy-makers and people work together to create a healthy future for adolescents.
